# Association of *Chlamydia Pneumoniae* Infection With Atherosclerotic Plaque Formation

**DOI:** 10.5539/gjhs.v8n4p260

**Published:** 2015-09-28

**Authors:** Omid Assar, Azim Nejatizadeh, Farzaneh Dehghan, Mohammad Kargar, Nader Zolghadri

**Affiliations:** 1 Alborz University of Medical Sciences, Karaj, Iran; 2 Cardiovascular Research Center, Hormozgan University of Medical Sciences, BandarAbbas, Iran; 3 Molecular Medicine Research Center, Hormozgan University of Medical Sciences, BandarAbbas, Iran; 4 Department of Microbiology, Islamic Azad University, Jahrom branch, Jahrom, Iran; 5 Department of Mathematics, Islamic Azad University, Sama College, BandarAbbas, Iran

**Keywords:** Chlamydia pneumoniae, atherosclerosis, nested polymerase chain reaction

## Abstract

Atherosclerosis is a complex multifactorial disorder. Studies show that infectious microbial agents may play an important role in the development of atherosclerosis; however, these findings are conflicting. This study investigated the presence of Chlamydia pneumoniae DNA in atherosclerotic plaques of patients suffering from coronary artery disease. In a cross-sectional study, 85 patients (43 females and 42 males with mean age of 61±9.5, range 42-82 years) referred for coronary artery bypass grafting (CABG) and thoracic biopsy as the control groups were enrolled for this study. Standard questionnaires, including demographic and clinical evaluation were administered. Obtained specimens were processed and then nested polymerase chain reaction with primers for Pst1 fragment was carried out to detect Chlamydia pneumoniae DNA. Statistical analysis was done using the SPSS software. Of note, in 25 out of the 85 patients (29.4%), C. pneumoniae was detected within atherosclerotic plaques, whereas, 5 out of the 85 thoracic biopsy (5.9%) were positive for the presence of the mentioned bacteria in internal thoracic artery. There was a statistically significant association between atherosclerotic plaque (study group) and thoracic biopsy (control group) in terms of C. pneumoniae positivity (P=0.0001). The findings of this study support the hypothesis that C. pneumoniae is associated with atherosclerosis.

## 1. Introduction

Inflammation plays an essential role in atherosclerotic plaque formation. Obviously, infections are proposed as strong determinants of atherosclerosis (Ross, 1999). It is evident that, infectious agents such as *C. pneumoniae* have the ability to initiate and develop a chronic inflammation as an infectious state (Kol, Sukhova, Lichtman, & Libby, 1998). Atherosclerosis is the leading cause of coronary vascular disease (CVD) (Izadi et al., 2013) which is a big challenge for the health status of humans (Jha & Mittal, 2013). Evidence indicates that morbidity and mortality of cardiovascular disease worldwide would be intensified from 28.9% in 1999 to 36.3% in 2020 (Selami, Suat, Ozcan, Serhat, & Turan, 2013). Development of athermanous plaques in coronary vessels of human seems to be multifactorial (Kwon et al., 2004). Although hypertension, hypercholesterolemia, smoking, diabetes mellitus, obesity are considered as the conventional risk factors for atherosclerosis. However, they could, in part, determine the pathogenesis of the disease (Kwon et al., 2004; Hedayat et al., 2009; Njamnshi et al., 2006; Al-Khatib & Al-Alawneh, 2013; Jha, Srivastava, Divya, Prasad, & Mittal, 2009).

The possible role of some organisms such as *C*. *pneumoniae, Helicobacter pylori, mycoplasma pneumoniae* and *herpes virus* family has been suggested in atherosclerotic disease (D.Pietro, Filardo, D. Santis, & Sessa, 2013; Rupp, Kothe, Mueller, Maass, & Dalhoff, 2003; Kaplan et al., 2006; Rupp & Maass, 2004). Among these organisms *C. pneumoniae* received the most attention, as it could be treated by antibiotic therapy (Sadeghian et al., 2013; Woods, Walker, McPherson, & Pincus, 2007; Zibaeenezhad, Amanat, Alborzi, & Obudi, 2005; Cherian, A.Bharati, Bobryshev, Nayar, & J. Ragavendra, 2006; Smieja, Mahony, Petrich, Boman, & Chernesky, 2002). In contrast, the causal role of *C. pneumoniae* infection remains to be elucidated (Loehe et al., 2002). *C. pneumoniae* is an obligatory intracellular bacterium that affects the majority of global adults and children during life (Peeling & Brunham, 1996). For the first time, in 1988, the association of this bacterium with coronary artery disease (CAD) was found (Saikku et al., 1988). Animal investigations were shown association between *C. pneumoniae* and development of atherosclerosis (Fong et al., 1997). Among the controversial studies, there is no consensus on the pathogenicity of *C. pneumoniae* in the initiation and formation of (Izadi et al., 2013) atherosclerotic plaque in humans.

*C. pneumoniae* infection and atherosclerotic heart disease are relatively high in Iran (Zibaeenezhad et al., 2005; Rostami et al., 2013; Shirani et al., 2006). It has been estimated that by 2030, more than 23 million people will die from cardiovascular disorders in the world (Jha & Mittal, 2013). In this regard, a study to investigate the presence of *C. pneumoniae* in atherosclerotic plaque of Iranian patients using polymerase chain reaction (PCR) was conducted. Furthermore, this study determined the correlation between the bacterium and atherosclerotic risk factors, in patients undergoing coronary artery bypass graft (CABG). It seems that the obtained results could help in elucidating the pathogenesis of atherosclerosis.

## 2. Methods and Materials

### 2.1 Study Population

In this cross-sectional study, 85 patients referred for coronary artery bypass grafting (CABG) at Jorjani Heart Center Of Shahid Mohammadi Hospital in Bandarabbas, from November 2010 to June 2011 were enrolled. None of the patients had previously undergone CABG or percutaneous coronary intervention. A standard questionnaire was used to collect demographic and clinical characteristics of patients which comprised of age, gender, obesity, body mass index (BMI) ≥ 30 kg/m^2^, smoking status (ex-smoker or current smoker), hypertension (reported history of hypertension or blood pressure ≥140 mmHg), hypercholesterolemia (total cholesterol>200 mg/dl and LDL>130 mg/dl), diabetes mellitus (fasting blood sugar level >126mg/dl or reported history of diabetes) and family history of coronary artery disease (Salarifar, Kazemeini,& Haji Zeinali, 2007) and complete history of prior medical conditions.

The ethics committee of the cardiovascular research center of Hormozgan University of medical sciences reviewed and approved the study. Written informed consents were obtained from the patients prior to specimen collection. Specimens from aortic atherosclerotic plaques (study group) and the same number was obtained from the internal mammary artery (control group) with no atherosclerotic degeneration were obtained during operation for coronary artery bypass grafting. All specimens were transported in Tris-EDTA buffer and kept at -80°C for DNA extraction and *C. pneumoniae* detection.

### 2.2 Molecular Analyses

DNA extraction from 25 mg of each specimen was performed using the Cinna Pure DNA extraction Kit (cinna gen, Iran), according to the manufacturer’s instructions. The extracted DNA was qualified by spectrophotometric method. Specific inner and outer primers reported by Reszka (Table 1) were used for amplification of *C. pneumoniae* *Pst1* fragment by nested PCR (Reszka, Jegier, Wasowicz, & Lelonek, 2008). The reactions were carried out in 25 μl of the reaction mixture containing 2 μl of DNA, 2.5 μl buffer 10x, 0.75 μl MgCl_2_ 50 mM, 0.4 μl dNTPs 10 mM, 0.5 μl of each outer primer at 10 pmol/μl and 0.25 μl of super Taq DNA polymerase (5U/μl). DNA was amplified during 40 cycles with an initial denaturation of 3 min at 95°C and a final extension of 5 min at 72°C. The cycling program consisted of 30 s denaturation at 95°C, 45 s annealing at 60°C and 30 s extension at 72°C. The reaction mixture of the second round PCR was identical to the first round, except that 1 μl of the first round product and inner primer (CPN1, CPN2) were used. Also, the PCR conditions were the same for the first round, but only 35 cycles were used. Negative control (distilled water) and positive control (genome of *C. pneumoniae*, vircell, Spanish) were run with all reactions. The products were visualized by electrophoresis on a 2% agarose gel with an appropriate molecular size marker (100bp DNA ladder, Gen Fanavaran, Iran (and examined under UV light.

**Table 1:** PCR primer for amplification of *C.pneumonia* *Pst1* fragment

Primer name	Primer sequence (5’-3’)	Size (bp)
CP1	TTATTCACCGTCCTACAGCAGAAA	404
CP2	GGGGGTTCAGGGATCATTTGT	
CPN1	TTACGAAACGGCATTACAACGGCTAGAAATCAAT	214
CPN2	TATGGCATATCCGCTTCGGGAACGAT	

### 2.3 Data Analysis

Statistical analysis was carried out by means of SPSS version 19 software program to compare categorical variables. Fisher’s Exact Test (2-sided P- value) was used. A P-value of less than 0.05 was considered statistically significant.

## 3. Results

**Figure 1:**
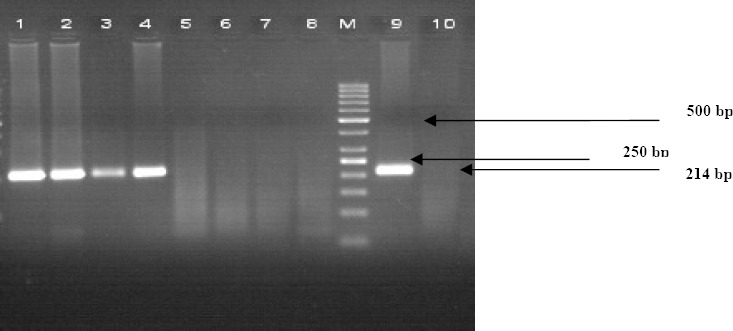
PCR product of C.pneumoniae was electrophoresed in 2% Agarose gel. Lane1-4 positive sample, 5-8 negative sample, M, size marker, 9, Positive control, 10, negative control

A total of 85 patients including 43 (50.6%) females and 42 (49.4%) males with a mean age of 61 years (range of 42-82) participated in the study. *C. pneumoniae* DNA (Figure 1) was positive in 29.4% (25 out of 85) of atherosclerotic plaques, whereas in samples of internal mammary artery biopsies, 5.9% (5 out of 85) were detected. Simultaneously, 3 patients were positive for *C. pneumoniae* in both atherosclerotic plaques and internal mammary artery biopsies. These results revealed a statistically significant association between *C. pneumoniae* DNA positivity in atherosclerotic plaques and atherosclerosis (P= 0.0001). Nine (9) out of the 85 patients had no atherosclerotic risk factor but other patients showed 1 to 5 risk factors. Demographic and clinical features in patient with *C. pneumoniae* positive and negative DNA are summarized in Table 2. There was no statistically significant difference between *C. pneumoniae* positive DNA and the atherosclerotic risk factors.

**Table 2:** Clinical Characteristics of Patient with respect to C. pneumoniae DNA Positivity

Parameters	*C.pneumoniae* in Aorta		*C.pneumoniae* in Mammary artery biopsy		P-value

	Positive n=25	Negative n=60	Positive n=5	Negative n=80	
Mean Age	60.3±9.7	61.7 ±9.5	63.6 ± 5.2	61.1 ± 9.7	0.12
Female	14 (%56)	29(%48.3)	4(%80)	39(%48.8)	0.55
Male	11 (%44)	31(%51.7)	1(%20)	40(%51.2)	
Smoking Habit	9(%36)	27(%45)	3(%60)	33(%41.2)	0.44
Diabetes	10(%40)	23(%38.3)	4(%80)	33(%41.2)	0.05
Hypertension	9(%36)	31(%51.7)	5(%100)	35(%43.8)	0.62
Hypercholesterolemia	5(%36)	31(%51.7)	5(%100)	35(%43.8)	0.86
Obesity	2(%8)	2(%3.3)	0(%0)	4(%5)	0.65
Family History	8(%32)	11(%18.3)	3(%60)	16(%20)	0.17

## 4. Discussion

Chronic infections and atherosclerosis showed unknown associations for years (Izadi et al., 2013. Izadi et al., 2014). C. pneumoniae is an intracellular obligatory microorganism (Wohlschlaeger, Wimmer, Nogler, Haberl, & Weis, 2005), and for the first time, Shore et al described the presence of *C. pneumoniae* in atheroma plaques, by the PCR method (Shore, Kuo,& Patton, 1992),. It has also been indicated as a possible cause of atherosclerosis (Joshi, Khandelwal, Joshi, & Gupta, 2013). Subsequently, researchers looked for a possible association between C. pneumoniae and atherosclerosis, because of the high prevalence of *C. pneumoniae* infection in the general population (Izadi et al., 2013). In this study, *C. pneumoniae* was detected with a relative frequency of 29.4% in atherosclerotic plaque and 5.9% in biopsies from the internal mammary artery and a significant difference was observed between *C. pneumoniae* and atherosclerosis.

The findings of this study correspond to studies conducted by Sessa et al. (2007), Lui et al. (2006), Petyaev et al. (2010), Chatzidimitriou et al. (Chatzidimitriou, Melidou, Gioula, Exindari, & Georgios, 2009), Kaklikkaya et al. (Kaklikkaya et al., 2006) and Wang et al. (Wang et al., 2007), whereas contrasting findings such as that of Bayram et al. (Bayram, Erdoğan, Ekşi, & Yamak, 2011), Know et al. (2004), Reszka et al. (Reszka, Jegier, Wasowicz, & Lelonek, 2008) and Iriz et al. (2008) succeeded to detect *C. pneumoniae* DNA in 30, 1.6, 27.5 and 15.1% of their studied samples respectively. However, these studies could not find any association between *C. pneumoniae* and atherosclerosis. Moreover, the studies of Tremolada et al. (2011), Bishara et al. (2003), Satpathy et al. (Satpathy, Bhan, Sharma, & Kar, 2008), Sodeck et al. (2004), West et al. (2009), Campbell et al. (Campell & Kuo, 2004), Apfalter et al. (2004), Watt et al. (Watt, Aesch, Lanotte, Tranquart, & Quentin, 2003) and Bossone et al. (2008) did not isolate *C. pneumoniae* DNA from atherosclerotic specimens by PCR. Furthermore, findings of the same studies in Iran with the association between *C. pneumoniae* infection and atherosclerosis are contrasting, where some studies revealed an association between *C. pneumoniae* infection and atherosclerosis (Rostami et al., 2013; Bahrmand et al., 2004; Dabiri et al., 2009) while, others could not (Sadeghian et al., 2013; Zibaeenezhad et al., 2005; Pooria et al., 2009; Hosseinian, Habibzadeh, Ahari, & Mokhtarpoor, 2007).

Disparity in the prevalence of *C. pneumoniae* could be due to selection of samples (Jha & Mittal, 2013), differences in the size of tested samples (Bishara et al., 2003) and using various methods such as serology, PCR, Immunocytochemistry, electron microscopy and culture (Forbes, Sahm,& Weissfeld., 2002) at diverse periods (Ieven & Hoyman, 2005).

Also, this study did not observe any significant difference in the atherosclerotic risk factors between the two patient groups in terms of *C. pneumoniae* PCR positive and *C. pneumoniae* PCR negative. In addition, the prevalence of these factors was lower in the *C. pneumoniae* positive group. Interestingly, 4 out of the 29 patients with positive PCR had none of the atherosclerotic risk factors while the others had one to five risk factors. It appears unlikely that infection seems to be the only or main cause of atherosclerosis (Dabiri et al., 2009).

The results of this study support the studies of Farsak et al from Turkey (Farsak et al., 2000) and Loehe et al from Germany (Loehe et al., 2002) but were different to that of Iran. Dabiri et al. in their study found high prevalence of *C. pneumoniae* infection among smokers than in non-smokers (Campell & Kuo, 2004). Furthermore, Eslami et al. and Hedayat et al. reported higher total Cholesterol in the PCR positive group (Pooria et al., 2009; Fallah et al., 2000). One possible explanation for this discrepancy is that, the number of male patients with higher incidence of heart disease and risk factors were more, while there was an approximately equal number of a male and female patient, likely influencing the results of the risk factors.

However, several limitations were encountered in the present study. This study could not consider the *C. pneumoniae* strains because the association of this chronic infection with the risk of atherosclerosis is likely limited to specific pathogenic strain. Furthermore, lack of serological information of the studied patients is another limitation of this study.

## 5. Conclusion

The results of this study support the hypothesis that *C. pneumoniae* is associated with atherosclerosis. Hence, efforts for eradication of *C. pneumoniae* infection are needed to determine whether its eradication decreases the incidence of this major life-threatening disease or not.
